# The clinicopathological and molecular characteristics of resected *EGFR*‐mutant lung adenocarcinoma

**DOI:** 10.1002/cam4.4543

**Published:** 2022-01-13

**Authors:** Wensheng Zhou, Zhichao Liu, Yanan Wang, Yanwei Zhang, Fangfei Qian, Jun Lu, Huimin Wang, Ping Gu, Minjuan Hu, Ya Chen, Zhengyu Yang, Ruiying Zhao, Yuqing Lou, Baohui Han, Wei Zhang

**Affiliations:** ^1^ Department of Pulmonary Medicine Shanghai Chest Hospital Shanghai Jiao Tong University Shanghai China; ^2^ School of Medicine Shanghai Jiao Tong University Shanghai China; ^3^ Department of Thoracic Surgery Shanghai Chest Hospital Shanghai Jiao Tong University Shanghai China; ^4^ Department of Pathology Shanghai Chest Hospital Shanghai Jiao Tong University Shanghai China

**Keywords:** concomitant mutation, *EGFR*, lung adenocarcinoma, recurrence‐free survival

## Abstract

**Background:**

Epidermal growth factor receptor (*EGFR*) mutations were frequently found with concomitant genetic alterations in lung adenocarcinoma (LUAD). This study aimed to investigate the profile of concomitant alterations of *EGFR*‐mutant LUAD ≤3 cm in size and its prognostic effect on recurrence.

**Methods:**

From January 2018 to December 2018, patients with resected LUAD ≤3 cm in size in Shanghai Chest Hospital were identified. All patients underwent capture‐based targeted next‐generation sequencing (NGS) with a panel of 68 lung cancer‐related genes and were found with *EGFR* mutation. Clinicopathological and molecular characteristics and recurrence‐free survival (RFS) were analyzed.

**Results:**

A total of 637 patients were enrolled in this study. The top three frequent co‐mutational genes were *TP53* (179 of 637, 28.1%), *PIK3CA* (27 of 637, 4.2%), and *ATM* (22 of 637, 3.5%). The most common amplified genes were *EGFR* (37 of 637, 5.8%), followed by *CDK4* (37 of 637, 5.8%) and *MYC* (12 of 637, 2.0%). Only *TP53* mutation and *EGFR* amplification were adverse prognostic factors for RFS (all *p *< 0.001) in univariate analysis. Multivariable analysis further demonstrated that *TP53* mutation and *EGFR* amplification were independent risk factors for RFS [(hazard ratio (HR) 2.07, 95% confidence interval (CI) 1.07–4.00, *p* = 0.030; HR 3.09, 95% CI 1.49–6.40, *p* = 0.002, respectively].

**Conclusions:**

Concomitant *TP53* mutation and *EGFR* amplification were poor prognostic factors for RFS in patients with *EGFR*‐mutant resected LUAD. Our findings provide valuable understanding of the impact of concurrent alterations and implication for better implementation of precision therapy for patients.

## Graphical abstract

In lung adenocarcinoma, epidermal growth factor receptor (EGFR) mutations were frequently found with concomitant genetic alterations of which the impact on resected EGFR‐mutant lung adenocarcinoma was infrequently reported.We retrospectively reviewed 637 patients and found that TP53 mutation and EGFR amplification were poor prognostic factors for recurrence‐free survival.

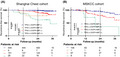

## 1 INTRODUCTION

Lung adenocarcinoma (LUAD) is the most common pathological subtype of lung cancer, which is the leading cause of cancer‐related mortality worldwide.^1^ For patients with early‐stage LUAD, surgery is the standard treatment. But even underwent radical resection, recurrence still takes place.

Previous studies explored the relationship between oncogene alteration and clinical outcome of early‐stage non‐small cell lung cancer (NSCLC). Epidermal growth factor receptor (*EGFR*) mutation represents the most common drugtable driver mutation in East Asian LUAD patients,^2^, ^3^ found as favorable prognostic factor.^4^ However, some studies showed that *EGFR* mutation is a negative prognostic indicator of recurrence‐free survival (RFS).^5^, ^6^ Intra‐tumor heterogeneity of LUAD lead to different biological behaviors, which may explain the inconsistent conclusions. Concomitant alterations reflected genetic characteristics of different clones in tumor, which were related to intra‐tumor heterogeneity. In advanced *EGFR*‐mutant LUAD, several studies revealed that concomitant alterations were associated with the efficacy of tyrosine‐kinase inhibitors (TKIs).^7^, ^8^ However, previous researches focused on advanced stage for targeted therapy and immunotherapy.^9^, ^10^ Only few studies have illustrated the concomitant mutations in *EGFR*‐mutant resected LUAD.^11^, ^12^, ^13^ Nowadays, next‐generation sequencing (NGS) was applied widely in clinical practice.^14^ Further research is thus needed to explore the association between gene alteration and RFS, to develop a more precise management after surgical resection.

In this study, we hypothesized that concurrent gene alterations have critical impact on RFS. In order to understand the clinicopathological and molecular characteristics in *EGFR*‐mutated patients, we performed this study to reveal the prevalence of *EGFR* concomitant alterations and their effect on RFS.

## 2 MATERIALS AND METHODS

### 2.1 Patients and sample collection

The study cohort consisted of 637 patients who underwent completely surgical resection and histologically confirmed with pathological size ≤3 cm LUAD at Shanghai Chest Hospital from January 2018 to December 2018 and was defined as Shanghai Chest cohort. All these patients have available NGS reports and confirmed with *EGFR*‐mutant status. The patients were staged based on the eighth edition of the International Association for the Study of Lung Cancer TNM classification for lung cancer.^15^

**FIGURE 1:**
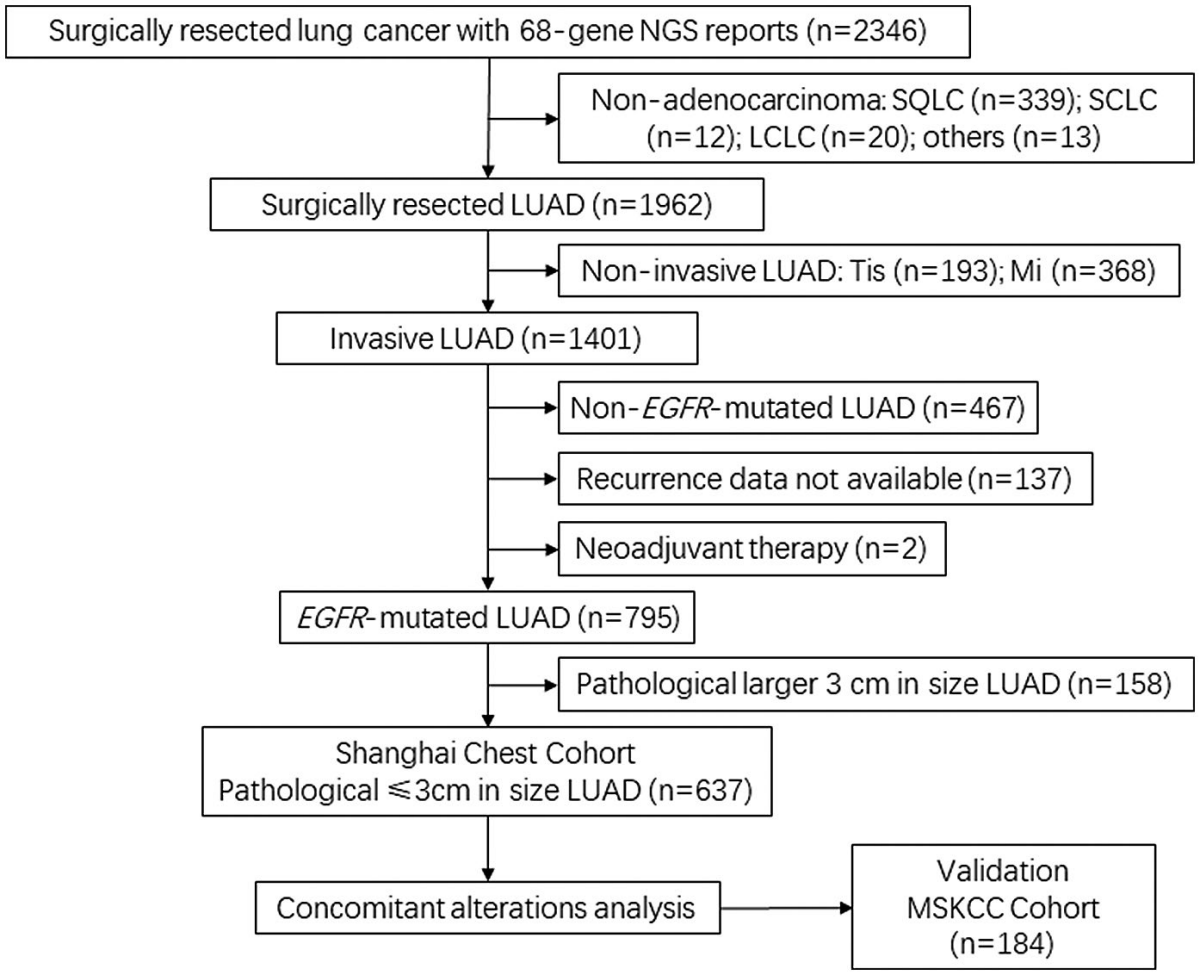
Workflow of current research. Abbreviations: NGS, next‐generation sequencing; SQLC, squamous cell lung cancer; SCLC, small cell lung cancer; LCLC, large cell lung cancer; LUAD, lung adenocarcinoma; Tis, tumor in situ; Mi, microinvasive carcinoma

Inclusion criterion were: (1) primary LUAD; (2) underwent completely surgical resection; (3) confirmed pathological size ≤3 cm; (4) all resected‐tissues were performed genetic analysis using 68‐gene NGS panel in Shanghai Chest Hospital; (5) NGS tests reported *EGFR* mutation positive (including exon 19 deletion, exon 21 L858R mutation, exon 20 insertion and exon 18 G719A mutation et al). Patients were excluded for: (1) non‐invasive LUAD (e.g., adenocarcinoma in situ, minimally invasive adenocarcinoma) or non‐adenocarcinoma; (2) preoperative neoadjuvant therapy. The study flowchart is shown in Figure 1.

The final follow‐up date was March 2021. Postoperative follow‐up was started the day the patient received surgery and performed every 3 months for the first 2 years, every 6 months for the next 2 years. The follow‐up data were obtained from hospital records or collected by telephone. RFS was calculated from the surgery date to recurrence or last follow‐up. All clinical data were collected from electronic records. The research was approved by the Research Ethics Board of Shanghai Chest Hospital and conducted in accordance with the Declaration of Helsinki as well, and it was deemed exempt from the requirement to gather participant consent by the Institutional Review Board (KS2039).

### 2.2 Targeted NGS

The DNA extraction was performed using the QIA amp DNA FFPE Tissue Kit (Qiagen, Hilden, Germany). Targeted NGS was performed to detect somatic mutations within each sample using a 68‐gene panel on the Nextseq500 sequencer (Illumina, Inc, Madison, WI, USA). The genomic profiles were assessed using Lung Core panel from Burning Rock Biotech (Guangzhou, China) (list of genes was provided in Table S1).

### 2.3 Sequence data analysis

Sequence data were mapped to the reference human genome (hg19) using Burrows‐Wheeler aligner v.0.7.10.^16^ Local alignment optimization, variant calling, and annotation were performed using Genome Analysis Tool Kit v.3.2 and VarScan.^17^, ^18^ Variants were filtered using the VarScan. Loci with depth less than 100 were filtered out. Minimal of five supporting reads were needed for INDELs and eight supporting reads were needed for SNV calling. According to the ExAC, 1000 Genomes, dbSNP, ESP6500SI‐V2 database, variants with population frequency over 0.1% were grouped as SNP and excluded from further analysis. Remaining variants were annotated with ANNOVAR.^19^

### 2.4 External cohort from cBioPortal database

The cBioPortal for Cancer Genomics (http://cbioportal.org/) is an open source for interactive exploration of multidimensional cancer genomic data that aims to translate data sets into biologic insights and clinical applications.^20^, ^21^ Patients with LUAD and available NGS as well as RFS data were identified in MSK‐IMPACT Clinical Sequencing Cohort (MSKCC, 2020; dataset ID: luad_mskcc_2020) as an external cohort for validation. Finally, 184 *EGFR*‐mutated patients were included in further analysis and defined as MSKCC cohort.

### 2.5 Statistical analysis

Fisher's exact test or Chi‐square test was used to compare the categorical data between two groups. RFS analysis was performed using the Kaplan‐Meier method and log‐rank test. Multivariable Cox proportional hazards model was applied to analyze factors correlating to RFS. SPSS (version 24.0, SPSS Inc, Chicago, IL, USA) and Prism software (version 8.0, GraphPad Software, San Diego, CA, USA) and R software (version 4.0.5, the R Foundation for Statistical Computing, Vienna, Austria) served for statistical analysis. Genes altered in 10 patients at least were considered. Gene amplification defined as copy number gains more than 2 times. Significant factors in univariable Cox proportional hazards model and factors with clinical significance were considered in multivariable Cox proportional hazards model. A *p* value <0.05 was considered to be statistically significant. Multiple testing was corrected by the false discovery rate (FDR) method on molecular variables’ univariable Cox analysis. The FDR was calculated by the p.adjust function derived from ‘Stats’ package in R software. The molecular variables with 2‐tailed *p* value <0.05 and FDR <0.05 were considered statistically significant with an acceptable FDR.

## 3 RESULTS

### 3.1 Baseline demographics

A total of 637 patients met the inclusion criteria and were enrolled for analysis. The clinicopathological and molecular characteristics of study cohort were shown in Table 1. In general,424 (66.6%) patients were female, 385 (60.4%) patients were older than 60 years and most of patients (92.3%) were never smokers. Two hundred and forty‐six (38.6%) patients were found with *EGFR* exon 19 deletion (19Del), while *EGFR* exon 21 L858R mutation (21L858R) was identified in 338 (53.1%) patients. The clinicopathological and molecular characteristics of MSKCC cohort can be found in Table S2.

**TABLE 1:** Clinicopathological and molecular characteristics of patients

	Total	%
Sex		
Male	213	33.4%
Female	424	66.6%
Age		
<60	252	39.6%
≥60	385	60.4%
Smoking		
Never	588	92.3%
Ever	49	7.7%
Tumor location		
Left	269	42.2%
Right	368	57.8%
Tumor size (cm)	1.84 ± 0.62	
T stage		
1a	54	8.5%
1b	328	51.5%
1c	168	26.4%
2a	60	9.4%
Other than 2a	27	4.2%
N stage		
0	583	91.5%
1	12	1.9%
2	42	6.6%
TNM stage		
IA	521	81.8%
IB	35	5.5%
IC	1	0.2%
IIA	0	0%
IIB	20	3.1%
IIIA	60	9.4%
Operation		
Lobectomy	461	72.4%
Segmentectomy	93	14.6%
Wedge resection	83	13.0%
High‐grade component predominant^a^		
No	609	95.6%
Yes	28	4.4%
VPI		
Absent	584	91.7%
Present	53	8.3%
Adjuvant chemotherapy		
No	575	90.3%
Yes	62	9.7%
*EGFR* mutation subtype		
19 Del	246	38.6%
21 L858R	338	53.1%
20ins and others	53	8.3%
*TP53*		
WT	458	71.9%
Mutant	179	28.1%
*EGFR* amplification		
No	600	94.2%
Yes	37	5.8%

Abbreviations: 19 Del, exon 19 deletion; 20ins, exon 20 insertion; 21 L858R, exon 21 L858R mutation; VPI, visceral‐pleural invasion; WT, wild type.

^a^ High‐grade component predominant was defined as micropapillary or solid pathological predominant subtype.

### 3.2 Somatic mutation and amplification of major genes

**FIGURE 2:**
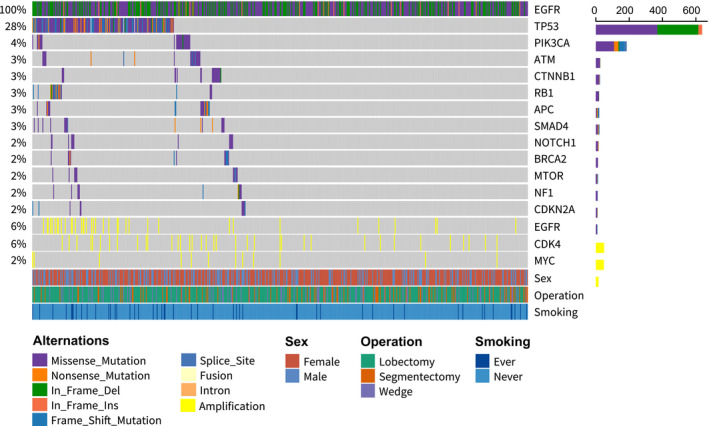
Concomitant alterations of Shanghai Chest cohort. Waterfall plot showing the alterations frequency of main genes in 637 *EGFR*‐mutant lung adenocarcinoma patients

The most frequent co‐mutational genes were *TP53* (179 of 637, 28.1%), followed by *PIK3CA* (27 of 637, 4.2%), *ATM* (22 of 637, 3.5%), *CTNNB1* (21 of 637, 3.3%), *RB1* (21 of 637, 3.3%), *APC* (20 of 637, 3.1%), *SMAD4* (17 of 637, 2.7%), *NOTCH1* (14 of 637, 2.2%), *BRCA2* (13 of 637, 2.0%), *MTOR* (12 of 637%,1.9%), *NF1* (11 of 637, 1.7%), *CDKN2A* (10 of 637, 1.6%). The most common amplification genes were *EGFR* (37 of 637, 5.8%), followed by *CDK4* (37 of 637, 5.8%), *MYC* (12 of 637, 2.0%). The overview of top 12 concurrent alterations and top 3 amplified genes are shown in Figure 2.

### 3.3 Comparison of characteristics between patients with or without TP53 mutation and EGFR amplification

To evaluate the clinical and pathological characteristics of co‐occurred *TP53* mutation or *EGFR* amplification in *EGFR*‐mutant patients, comparisons were summarized. *TP53* mutation was associated with cigarette exposure, larger tumor size, higher TNM stage, high‐grade‐component predominance and visceral pleural invasion (VPI). And the presence of *EGFR* amplification was associated with larger tumor size, higher TNM stage, high‐grade‐component predominance and VPI (Table S3).

The association of *TP53* mutation and *EGFR* amplification in *EGFR*‐mutated patients was also explored, showing that the presence of *EGFR* amplification was significantly associated with mutations in *TP53* in both cohorts (Figure S1). And the prevalence of *EGFR* amplification showed no difference between study cohort and external cohort [5.81% (37/637) vs. 4.89% (9/184), *p *= 0.719].

According to concomitant status of gene alterations, four groups of patients can be identified: (1) *EGFR* mutation only, (2) concomitant *TP53* mutation only, (3) concomitant *EGFR* amplification only, and (4) concomitant *TP53* and *EGFR* amplification. The analysis of tumor mutation burden and *EGFR* copy number gains in patients with *EGFR* amplification was showed in Figures S2 and S3, respectively.

### 3.4 Prognostic value of concurrent TP53 mutations or EGFR amplification in EGFR‐mutant patients

The median follow‐up duration was 30.57 months (interquartile range, 27.87–32.40). A total of 49 patients (7.7%) experienced recurrences. Survival analyses were performed and demonstrated the prognostic value of concomitant *TP53* mutation or *EGFR* amplification in *EGFR*‐mutant patients (Table S4).

**FIGURE 3:**
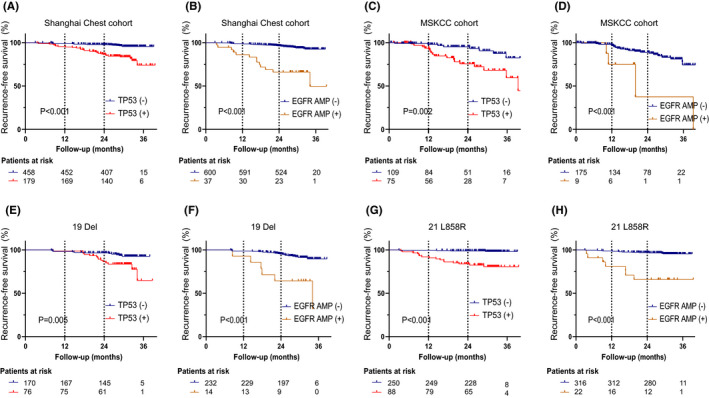
Kaplan–Meier survival curves of RFS for patients with and without *TP53* mutation and patients with and without *EGFR* amplification in Shanghai Chest cohort (A, B), in MSKCC cohort (C, D), *EGFR* 19 Del subgroup (E, F) and *EGFR* 21 L858R subgroup (G, H) in Shanghai Chest cohort. Abbreviations: RFS, recurrence‐free survival; 19 Del, exon 19 deletion; 21 L858R, exon 21 L858R mutation

Compared with patients harboring *TP53* WT (wild type), patients harboring *TP53* mutation had a significant worse RFS in Shanghai Chest cohort (*p *< 0.001; Figure 3A). Compared with patients with *EGFR* mutation only, patients with both *EGFR* mutation and amplification had a significant worse RFS (*p *< 0.001; Figure 3B). Similar results were observed in the external MSKCC cohort (*p* = 0.002, *p *< 0.001, respectively) in Figure 3C,D. Furthermore, in different mutation subtypes of *EGFR* (19Del and L858R), similar findings were also observed (*p* = 0.005, *p *< 0.001, *p *< 0.001, *p *< 0.001, respectively; Figure 3E–H).

### 3.5 Concomitant TP53 mutations or EGFR amplification is an independent prognostic factor of patients with EGFR‐mutant lung adenocarcinoma

Survival was analyzed using univariable and multivariable Cox proportional hazard regress model for RFS (Table 2). Univariable analysis revealed that TNM stage, high‐grade component predominant, *TP53* mutation and *EGFR* amplification were significant prognostic factors for RFS. Multivariable analysis further demonstrated that concurrent *TP53* mutation (HR 2.07, 95% CI 1.07–4.00, *p* = 0.030) and *EGFR* amplification (HR 3.09, 95% CI 1.49–6.40, *p* = 0.002) were independent adverse factors for RFS.

**TABLE 2:** Univariable and multivariable analysis of factors associated with recurrence‐free survival for resected *EGFR*‐mutated adenocarcinoma patients using Cox proportional hazard regression model (*N* = 637)

Variable	Univariate analysis			Multivariate analysis		
	HR	95%CI	*p* value	HR	95%CI	*p* value
Sex						
Female	reference					
Male	0.86	0.48–1.54	0.614			
Age						
<60	reference					
≥60	0.88	0.5–1.55	0.656			
Smoking status^a^						
Never	reference					
Ever	1.97	0.88–4.38	0.098	0.80	0.34–1.90	0.618
Tumor Size (cm)	2.39	1.51–3.79	<**0.001**	1.12	0.67–1.88	0.665
Tumor Location						
Left	reference					
Right	1.38	0.77–2.49	0.282			
TNM stage						
I	reference					
II+III	10.79	6.13–18.97	<**0.001**	5.41	2.67–10.95	**<0.001**
*EGFR* mutation subtype			0.212			
19 Del	reference					
21 L858R	0.59	0.33–1.06	0.079			
Others	0.72	0.25–2.08	0.547			
High grade component predominant						
No	reference					
Yes	8.04	4.09–15.81	<**0.001**	2.56	1.25–5.24	**0.010**
Operation						
Lobectomy	reference					
Segmentectomy/wedge resection	2.79	1.19–6.56	**0.019**	1.37	0.55–3.44	0.499
Adjuvant chemotherapy						
No	reference					
Yes	7.17	4.06–12.68	**<0.001**	1.33	0.61–2.92	0.477
*TP53* mutation						
WT	reference					
Mutant	4.73	2.65–8.46	**<0.001**	2.07	1.07–4.00	**0.030**
*EGFR* Amplification						
No	reference					
Yes	7.26	3.85–13.71	**<0.001**	3.09	1.49–6.40	**0.002**

Abbreviations: 19 Del, exon 19 deletion; 20ins, exon 20 insertion; 21 L58R, exon 21 L858R mutation; CI, confidence interval; HR, hazard ratio; WT, wild type.

^a^ Smoking status was considered as clinically significant factor and included in multivariable analysis.

The bold values were statistically significant.

**FIGURE 4:**
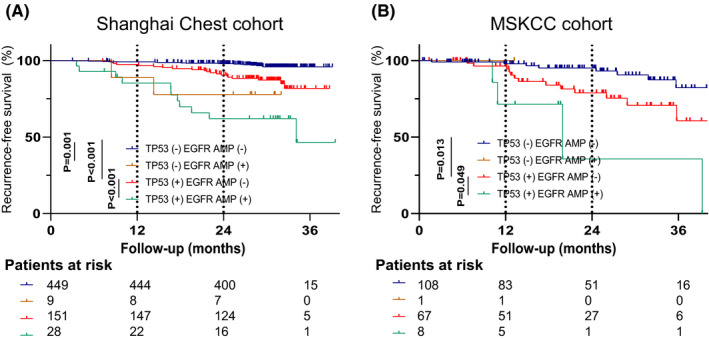
Kaplan–Meier survival curves of RFS for patients with no mutation in *TP53* or no amplification in *EGFR*, mutations in *TP53* alone, amplification in *EGFR* alone and both mutations in *TP53* and amplification in *EGFR* in Shanghai Chest cohort (A) and in MSKCC cohort (B). Abbreviations: RFS, recurrence‐free survival

Compared with others, the patients harboring *EGFR* amplification had a significant worse RFS, regardless of *TP53* status. (Figure 4A). Similarly, we found that patients with concomitant *TP53* mutation and *EGFR* amplification in the external MSKCC cohort had poorer RFS (Figure 4B).

The prognosis values of *TP53* mutation and *EGFR* amplification in RFS were also estimated by nomogram (Figure S4) and validated by calibration curves (Figure S5). Subgroup analyses of stage IA and stage IB‐IIIA were performed (Figure S6).

## 4 DISCUSSION

Recurrence is a critical problem in postoperative management of LUAD. Therefore, recurrence risk stratification is vital for identifying those who might benefit from more intensive adjuvant treatment for resected LUAD. *EGFR* is reported as the most frequent altered driver gene in Asian patients.^3^ Concomitant alterations are frequently noticed in LUAD. However, their prognostic role remains unclear in early‐stage LUAD. In the current study, we analyzed the data of 637 *EGFR*‐mutated patients and explored the prognostic value of concomitant alterations on recurrence. In order to minimize the impact of surgical approach and dissection extent and the effect of tumor's burden in primary site on prognosis, only small‐size (≤3 cm) cases were included in our study. Our study demonstrated that concomitant *TP53* mutations and *EGFR* amplification were poor prognostic factors for RFS in patients with *EGFR*‐mutant resected LUAD, indicating that early interventions may be considered in these patients. To our knowledge, this is the largest study that comprehensively focused on both *TP53* mutation and *EGFR* amplification in resected *EGFR*‐mutant patients.

*TP53*, functioning critically in cell cycle, DNA repair and metabolism,^22^ is the most common tumor suppressor gene in *EGFR*‐mutated lung adenocarcinoma.^10^, ^13^ Mutation in *TP53* gene was reported as poor prognostic predictor of EGFR‐TKIs treatment in advanced LUAD.^10^ Zhao et al^13^ analyzed 409 *EGFR*‐mutated patients and confirmed that patients with concurrent *TP53* mutation have worse (disease‐free survival) DFS, and Long et al^11^ as well as Lee et al^23^ showed the similar observations. In the current study, we found that 28.1% of patients carrying *TP53* mutation, which is similar to previous researches focusing on surgical *EGFR*‐mutated patients (15.83–53.54%). Moreover, *TP53* mutation was associated with aggressive clinicopathological features as previously reported.^24^ The survival analysis of both two cohorts further demonstrated that patients with concurrent *TP53* mutation have poorer RFS. In addition, mutations in *TP53* occur as early truncal events in tumor evolution and allow tolerance of a greater degree of genomic instability, resulting subclonal diversification and intra‐tumor,^25^, ^26^ which correlated with aggressively biological behaviors and higher tumor mutation burden (TMB). These findings indicated that *TP53*‐mutant status was an independent prognostic factor in *EGFR*‐mutant LUAD. As such, the postoperative management of LUAD should consider the mutation not only in *EGFR* but also in *TP53*, given its negative impact.

*EGFR* amplification occurred usually in *EGFR*‐mutant patients. Some studies showed that *EGFR* amplification is one of the resistance mechanisms of the third generation EGFR‐TKIs treatment, which lead to worse survival benefits in advanced patients.^27^, ^28^, ^29^ But in gefitinib‐treated studies, patients with *EGFR* amplification had better progression‐free survival than those without *EGFR* amplification.^30^, ^31^ However, there remains no study focusing on the impact of *EGFR* amplification in *EGFR*‐mutated surgical resected LUAD. In current research, 5.8% of patients have concurrent *EGFR* amplification. We investigated the characteristics of *EGFR* amplification in postoperative patients with *EGFR* mutation and explored the impact to RFS. Patients harboring *EGFR* amplification had worse RFS, regardless of *EGFR* mutation subtype. Possible reason may be that *EGFR* amplification was associated with increased mutant allele transcription and gene activity on the basis that mutation of *EGFR* activated receptor tyrosine kinase (RTK) pathway, cooperating with tumorigenesis and resulting in aggressive characteristics.^32^

Cancer develops through a process of somatic evolution.^33^ The association between *TP53* mutation and *EGFR* amplification may be complex. Previous research revealed that mutations in *TP53* lead to genetic instability and result in focal high‐amplitude amplifications that occur late during the evolution of lung cancer.^34^ Zhang et al.^35^ also reported that *EGFR* copy number gains occurred relatively late compared with *EGFR* mutation and *TP53* mutation in molecular time scale. In our search, the presence of *EGFR* amplification was correlated with *TP53* mutation in both two cohorts, which indicated *EGFR* amplification arise relatively late and toward the end of the evolution of *EGFR*‐mutated adenocarcinoma, resulting in aggressive pathological characteristics (e.g., high‐grade‐component predominance and lymphatic metastases). Therefore, patients with *EGFR* amplification should be regarded as high recurrence‐risk population in *EGFR*‐mutated patients.

According to previous researches, mutation in *TP53* correlated with higher TMB.^36^, ^37^ TMB significantly distinguished the patients with inferior RFS from entire MSKCC cohort, but there was no difference in TMB between tumors with *TP53* mutation and those with *TP53* mutation and *EGFR* amplification concurrently, indicating that the reasons were likely to be that TMB is not the major prognostic factor in patients with *TP53* mutation. The inner mechanism of the inferior RFS of patients with concomitant *TP53* and *EGFR* amplification is complex and needed to be explored in further studies.

The analysis of *EGFR* copy number displayed that *EGFR* amplification was significantly associated with recurrence, while *EGFR* copy number showed no different between recurrent patients and non‐recurrent patients. In addition, patients with lower copy number has no better survival than those with higher copy number. It suggested that the status of *EGFR* amplification happening may weight more important than its frequency (copy number); therefore, further mechanism account for this result still needs future exploration.

With the improvement of early lung cancer scanning, the proportion of surgical lung cancer patients is increasing,^38^ and *EGFR*‐mutate patients are the major part of them especially in Asia.^2^ For surgical resected *EGFR*‐mutated patients, monitoring the recurrence of tumor is performed currently according to clinicopathologic risk stratification, which could be improved in some way. In recent years, the development of adjuvant therapy was promoted due to the clinical trials focusing on the use of EGFR‐TKIs in post‐operative management.^39^, ^40^, ^41^ ADUARA trial on adjuvant EGFR‐TKIs revealed that even the stage IB patients can benefit from adjuvant EGFR‐TKIs, which indicated that the better postoperative management is needed for *EGFR*‐mutated patients.^40^ Providing predictive and prognostic values in advanced cancer treatment, NGS was used to guide clinical practice. Genomic alterations stratified the treatment‐benefit cohort in targeted therapy and immunotherapy,^42^ which confirms the clinical value of genetic alterations. Risk stratification by molecular features can help to differentiate the high‐risk *EGFR*‐mutant patients from low‐risk *EGFR*‐mutated patients. This study contributed to uncovering the risk cohorts according to molecular risk stratification, which may benefit more from earlier and more intensive adjuvant therapy. There is no research focusing on the postoperative management of these specific population, but researches focusing on advanced NSCLC patients have showed that *EGFR* amplification was associated with treatment guiding benefits. The subgroup analyses of IPASS trial were reported that PFS favored gefitinib over carboplatin/paclitaxel in *EGFR*‐mutated patients with high *EGFR* copy number.^43^ A. Ruiz‐Patiño et al. and Cui J et al. had reported that *EGFR* amplification was associated with better survival when treated with EGFR‐TKIs.^30^, ^44^ Additionally, among patients with *EGFR* amplification, high or low copy number did not affect the treatment outcomes.^44^ However, *EGFR* amplification was also reported as one of the resistance mechanisms of 3^rd^ generation EGFR‐TKIs,^45^ which may indicated that patients with *EGFR* amplification benefit less from 3^rd^ generation EGFR‐TKIs therapy, compared to patients without *EGFR* amplification. But there is no research comparing the clinical outcomes of being treated with different generation EGFR‐TKIs in patients with *EGFR* amplification, which should be explored in further research. Moreover, 1^st^ plus 3^rd^ generation EGFR‐TKIs was designed following biomarkers strategy to overcome the resistance of 3^rd^ generation TKIs (*EGFR* amplification) in the phase II ORCHARD trial,^46^ which will provide valuable guidance for these specific population.

Our study has several limitations. Selection bias is inevitable for single‐center retrospective study. The frequency of different mutation subtypes in *EGFR* was similar with previous researches,^4^, ^5^ which may indicate the minor selection bias. The number of *EGFR* amplification events was small, but there was no statistically significant difference in frequency of *EGFR* amplification between Shanghai Chest cohort and MSKCC cohort. Postoperative NGS detection was performed widely since 2018 in our institution. Long‐term follow‐up is needed for more conclusive statements. What's more, the mechanism should be explored in experiment research.

## 5 CONCLUSIONS

Concomitant *TP53* mutation and *EGFR* amplification were independent adverse factors for RFS in patients with *EGFR*‐mutant resected LUAD. Our findings provide valuable understanding of the impact of concurrent alterations and implication for better implementation of precision therapy for these patients.

## ETHICAL APPROVAL STATEMENT

Data are collected from Shanghai Chest Hospital and are devoid of any personal identifiable information (KS2039).

## Conflict of Interest

The authors have no conflicts of interest to declare.

## AUTHOR CONTRIBUTIONS

Wensheng Zhou: Conceptualization, Data curation, Formal analysis, Project administration, Software, Writing ‐ original draft, Writing ‐ review & editing. Zhichao Liu: Data curation, Formal analysis, Investigation, Methodology, Software. Yanan Wang: Investigation, Software, Writing ‐ review & editing. Yanwei Zhang: Project administration, Methodology. Fangfei Qian: Software, Formal analysis. Jun Lu: Software, Formal analysis, Data curation. Huimin Wang: Data curation, Investigation, Project administration. Ping Gu: Software, Project administration. Minjuan Hu: Data curation, Software. Ya Chen: Data curation, Investigation. Zhenyu Yang: Data curation, Investigation. Ruiying Zhao: Software, Formal analysis. Yuqing Lou: Supervision, Validation, Visualization, Writing – review. Baohui Han: Conceptualization, Supervision, Writing ‐ review & editing. Wei Zhang: Conceptualization, Project administration, Supervision, Validation, Visualization, Writing ‐ review & editing. All authors reviewed and approved the final version of the manuscript.

## Supporting information

Figure S1

Figure S2

Figure S3

Figure S4

Figure S5

Figure S6

Table S1‐S4

## Data Availability

The datasets used and analyzed during the current study are available from the corresponding author on reasonable request.
