# Supplementary material for: The clinicopathological and molecular characteristics of resected EGFR‐mutant lung adenocarcinoma
Source: Cancer Med. 2022 Jan 13;11(5):1299–309. doi: 10.1002/cam4.4543 (PMC8894712; doi:10.1002/cam4.4543)
Supplement: Supplementary file 7 — Table S1‐S4 [file CAM4-11-1299-s001.docx]

**Supplementary Figure legends**

Supplementary Figure S1 Frequency of *EGFR* amplification in two cohorts.

Supplementary Figure S2 (A) Tumor mutation burden (TMB) analyses among subgroups in MSKCC cohort; the Kaplan-Meier plot depicting recurrence-free survival (RFS) in all patients with *EGFR* mutation (B), patents with concomitant *TP53* mutation(C) and patients with *EGFR* amplification (D) stratified by TMB (high/low). Cutoffs were the median of TMB in each subgroup; (E) Correlation analysis between TMB and RFS in patient with *EGFR* amplification.

Supplementary Figure S3 *EGFR* copy number analyses in Shanghai Chest cohort (A) the frequency of *EGFR* amplification with respect to recurrence in entire cohort; (B) the comparison of *EGFR* copy number between patients with and without recurrence; (C) the correlation analysis between *EGFR* copy number and recurrence-free survival (RFS) in patients with *EGFR* amplification; (D) Kaplan-Meier plot depicting RFS in patients with *EGFR* amplification stratified by *EGFR* copy number gains (high/low). Cutoff was the median of *EGFR* copy number.

Supplementary Figure S4 Nomogram for 1-, 2-year recurrence survival in *EGFR* mutate patients based on multi-variable analysis in Shanghai Chest cohort. Example: case with Stage II, *EGFR* mutation positive as well as *TP53* mutation and *EGFR* amplification.

Supplementary Figure S5 Calibration curves of nomogram for predicting 1-，2-year RFS in Shanghai Chest cohort (A and B) and in MSKCC cohort (C and D).

Supplementary Figure S6 Subgroup analyses of *TP53* mutation and *EGFR* amplification in stage IA (A and B) and stage IB-IIIA patients (C and D).

Supplementary Table 1. Gene list of NGS Panel

| 68-gene panel list |  |
| --- | --- |
|  | ALK BRAF KRAS MET AKT1 APC ATM AXL ERBB3 ESR1  FGF3 FGFR3 IDH2 KDR MYC NRG1 STK11 TSC2 FGF4 FLT3  EGFR RET AR BRCA2 CCND1 CD74 CDK6 ERBB2 ROS1 ARAF  BCL2L11 BRCA1 CDK4 CDKN2A CTNNB1 DDR2 FGF19 FGFR2  IDH1 JAK2 IFG1R KIT NF1 RB1 ERBB4 FGFR1 HRAS JAK1 MAP2K1  MTOR NOTCH1 NRAS PDGFRA PIK3CA PTCH1 RAF1 TOP2A TP53  CYP2D6 DPYD NTRK1 NTRK2 NTRK3 PTEN SMAD4 SMO TSC1 UGT1A1 |

Abbreviations: NGS = next-generation sequencing.

Supplementary Table 2 Clinicopathologic and molecular characteristics of patients in MSKCC cohort

|  | Total | % |
| --- | --- | --- |
| **Sex** |  |  |
| Male | 45 | 24.5% |
| Female | 139 | 75.5% |
| **Age** |  |  |
| ＜65 | 74 | 40.2% |
| ≥65 | 110 | 59.8% |
| **Smoking** |  |  |
| Never | 87 | 47.3% |
| Ever | 97 | 52.7% |
| **Tumor Size (cm)** | 2.18 ± 1.00 | |
| **TMB (mut/Mb)** | 4.70 ± 6.57 | |
| **TNM stage** |  |  |
| 1 | 144 | 78.3% |
| 2 | 20 | 10.9% |
| 3 | 20 | 10.9% |
| **N metastasis** |  |  |
| No | 154 | 83.7% |
| Yes | 30 | 16.3% |
| **High-grade component predominant** |  |  |
| No | 165 | 89.7% |
| Yes | 19 | 10.3% |
| ***EGFR* mutation subtype** |  |  |
| 19 Del | 64 | 34.8% |
| 21 L858R | 83 | 45.1% |
| 20ins and others | 37 | 20.1% |
| ***TP53*** |  |  |
| WT | 109 | 59.2% |
| mutant | 75 | 40.8% |
| ***TP53* mutation subtype** |  |  |
| Missense Mutation | 48 | 26.1% |
| Nonsense Mutation | 11 | 6.0% |
| Frameshift Mutation | 8 | 4.3% |
| Others | 8 | 4.3% |
| ***EGFR* amplification** |  |  |
| No | 175 | 95.1% |
| Yes | 9 | 4.9% |

Abbreviations: TMB = tumor mutation burden; 19 Del = exon 19 deletion; 21 L58R = exon 21 L858R mutation; 20ins = exon 20 insertion.

Supplementary Table 3. Clinical and pathological characteristics of *EGFR*-mutant patients with or without *TP53* mutation or *EGFR* amplification in Shanghai Chest cohort

|  | *TP53* | | | | P value | *EGFR* Amplification | | | | P value |
| --- | --- | --- | --- | --- | --- | --- | --- | --- | --- | --- |
|  | WT | % | Mutant | % |  | WT | % | Mutant | % |  |
| Sex |  |  |  |  |  |  |  |  |  |  |
| Male | 146 | 31.9% | 67 | 37.4% | 0.192 | 200 | 33.3% | 13 | 35.1% | 0.858 |
| Female | 312 | 68.1% | 112 | 62.6% |  | 400 | 66.7% | 24 | 64.9% |  |
| Age |  |  |  |  |  |  |  |  |  |  |
| ＜60 | 175 | 38.20% | 77 | 43.0% | 0.280 | 235 | 39.20% | 17 | 45.90% | 0.489 |
| ≥60 | 283 | 61.80% | 102 | 57.0% |  | 365 | 60.80% | 20 | 54.10% |  |
| **Smoking status** |  |  |  |  |  |  |  |  |  |  |
| Never | 432 | 94.3% | 156 | 87.2% | **0.004** | 557 | 92.8% | 31 | 83.8% | **0.056** |
| Ever | 26 | 5.7% | 23 | 12.8% |  | 43 | 7.2% | 6 | 16.2% |  |
| Tumor Location |  |  |  |  |  |  |  |  |  |  |
| Left | 197 | 43.0% | 72 | 40.2% | 0.534 | 252 | 42.0% | 17 | 45.9% | 0.732 |
| Right | 261 | 57.0% | 107 | 59.8% |  | 348 | 58.0% | 20 | 54.1% |  |
| **Tumor Size (cm)** | 1.80±0.61 | | 1.97±0.64 | | **0.001** | 1.82±0.61 | | 2.25±0.62 | | **＜0.001** |
| **T stage** |  |  |  |  |  |  |  |  |  |  |
| 1a | 41 | 9.0% | 13 | 7.3% | **＜0.001** | 53 | 8.8% | 1 | 2.7% | **＜0.001** |
| 1b | 260 | 56.8% | 68 | 38.0% |  | 318 | 53.0% | 10 | 27.0% |  |
| 1c | 113 | 24.7% | 55 | 30.7% |  | 153 | 25.5% | 15 | 40.5% |  |
| 2a | 25 | 5.5% | 35 | 19.6% |  | 49 | 8.2% | 11 | 29.7% |  |
| Other than 2a | 19 | 4.1% | 8 | 4.5% |  | 27 | 4.5% | 0 | 0.0% |  |
| **N stage** |  |  |  |  |  |  |  |  |  |  |
| 0 | 437 | 95.4% | 146 | 81.6% | **＜0.001** | 557 | 92.8% | 26 | 70.3% | **＜0.001** |
| 1 | 7 | 1.5% | 5 | 2.8% |  | 9 | 1.5% | 3 | 8.1% |  |
| 2 | 14 | 3.1% | 28 | 15.6% |  | 33 | 5.7% | 8 | 21.6% |  |
| **TNM stage** |  |  |  |  |  |  |  |  |  |  |
| I | 419 | 91.5% | 138 | 77.1% | **＜0.001** | 531 | 88.5% | 26 | 70.3% | **0.003** |
| II+III | 39 | 8.5% | 41 | 22.9% |  | 17 | 11.5% | 3 | 29.7% |  |
| **Operation** |  |  |  |  |  |  |  |  |  |  |
| lobectomy | 319 | 69.7% | 142 | 79.3% | **0.044** | 428 | 71.3% | 33 | 89.2% | **0.007** |
| Segmentectomy | 75 | 16.4% | 18 | 10.1% |  | 93 | 15.5% | 0 | 0.0% |  |
| wedge resection | 64 | 14.0% | 19 | 10.6% |  | 79 | 13.2% | 4 | 10.8% |  |
| **High-grade component predominant^†^** |  |  |  |  |  |  |  |  |  |  |
| No | 446 | 97.4% | 163 | 91.1% | **0.001** | 578 | 96.3% | 31 | 83.8% | **0.004** |
| Yes | 12 | 2.6% | 16 | 8.9% |  | 22 | 3.7% | 6 | 16.2% |  |
| **VPI** |  |  |  |  |  |  |  |  |  |  |
| absent | 437 | 95.4% | 147 | 82.1% | **＜0.001** | 556 | 92.7% | 28 | 75.7% | **0.002** |
| present | 21 | 4.6% | 32 | 17.9% |  | 44 | 7.3% | 9 | 24.3% |  |
| EGFR mutation subtype |  |  |  |  |  |  |  |  |  |  |
| 19 Del | 170 | 37.1% | 76 | 42.5% | 0.4292 | 232 | 38.70% | 14 | 37.80% | 0.5031 |
| 21 L858R | 250 | 54.60% | 8800.0% | 49.20% |  | 316 | 52.70% | 22 | 59.50% |  |
| 20ins and others | 38 | 8.3% | 15 | 8.40% |  | 52 | 8.70% | 1 | 2.70% |  |
| **Adjuvant chemotherapy** |  |  |  |  |  |  |  |  |  |  |
| No | 431 | 94.1% | 144 | 80.4% | **＜0.001** | 549 | 91.5% | 26 | 70.3% | **＜0.001** |
| Yes | 27 | 5.9% | 35 | 19.6% |  | 51 | 8.5% | 11 | 29.7% |  |

^†^High-grade component predominant was defined as micropapillary or solid pathological predominant subtype.

Abbreviations: WT = wild type; VPI = visceral pleural invasion; 19 Del = exon 19 deletion; 21 L58R = exon 21 L858R mutation; 20ins = exon 20 insertion.

Supplementary Table 4. Univariable analysis for altered genes.

| Gene | HR | 95.0% CI | P value | FDR |  |
| --- | --- | --- | --- | --- | --- |
|  |  |  |  |  |  |
| *TP53* | 4.73 | 2.65-8.46 | ＜0.001 | **＜0.001** |  |
| *PIK3CA* | 1.56 | 0.49-5.02 | 0.455 | 0.84 |  |
| *ATM* | 0.56 | 0.08-4.09 | 0.571 | 0.857 |  |
| *RB1* | 2.81 | 1.01-7.83 | 0.047 | 0.141 |  |
| *CTNNB1* | 1.22 | 0.3-5.03 | 0.783 | 0.903 |  |
| *APC* | 2.1 | 0.65-6.76 | 0.213 | 0.456 |  |
| *SMAD4* | 1.62 | 0.39-6.68 | 0.504 | 0.84 |  |
| *NOTCH1* | 3.66 | 1.14-11.79 | 0.03 | 0.141 |  |
| *BRCA2* | 1.02 | 0.14-7.38 | 0.986 | 0.996 |  |
| *MTOR* | 0.7 | 0.1-5.04 | 0.72 | 0.903 |  |
| *NF1* | 0 | 0-Inf | 0.996 | 0.996 |  |
| *CDKN2A* | 1.32 | 0.18-9.61 | 0.783 | 0.903 |  |
| *EGFR* AMP | 7.26 | 3.85-13.71 | ＜0.001 | **＜0.001** |  |
| *CDK4* AMP | 1.92 | 0.76-4.84 | 0.167 | 0.4175 |  |
| *MYC* AMP | 3.31 | 1.03-10.66 | 0.045 | 0.141 |  |

Abbreviations: AMP = amplification; HR = hazard ratio; CI = confidence interval; FDR = false discovery rate.
